# Supplementary material for: Renal Proximal Tubule Cell Cannabinoid-1 Receptor Regulates Bone Remodeling and Mass via a Kidney-to-Bone Axis
Source: Cells. 2021 Feb 17;10(2):414. doi: 10.3390/cells10020414 (PMC7922053; doi:10.3390/cells10020414)
Supplement: Supplementary file 1 [file cells-10-00414-s001.pdf]

cells

MDPI

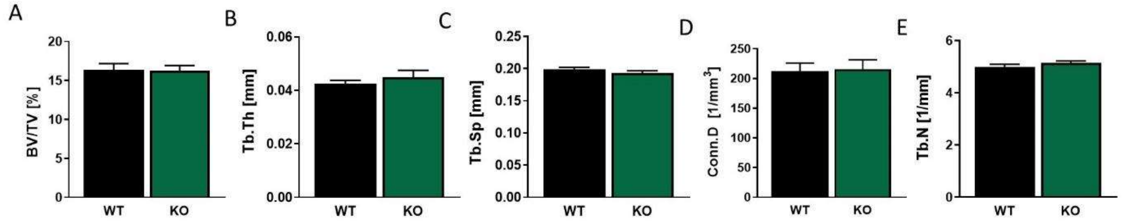

Supplementary Figure 1. Effect of CB_1R nullification in RPTCs on vertebral parameters. Analysis of bone volume density of L3 vertebrae (BV/TV) values (**A**), L3 Trabecular thickness (Tb.Th; **B**), L3 Trabecular spacing (Tb.Sp; **C**), L3 Connectivity density (Conn.D; **D**), and L3 Trabecular number (Tb.N; **D**). Data represent the mean ± SEM from 10-14 animals per group.

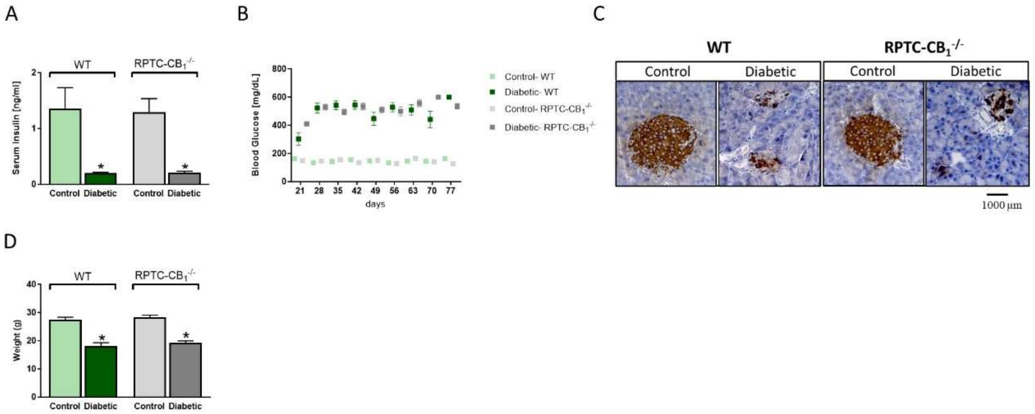

Supplementary Figure 2. Induction of T1D in mice lacking CB$_1$R in RPTCs by using STZ. Serum insulin levels (**A**), Blood glucose (**B**), Representative insulin staining of the pancreas from each treatment group (**C**), and body weight (**D**). Data represent the mean ± SEM from 8-13 animals per group. *P<0.05 vs. non-diabetic control mice in each mouse strain.

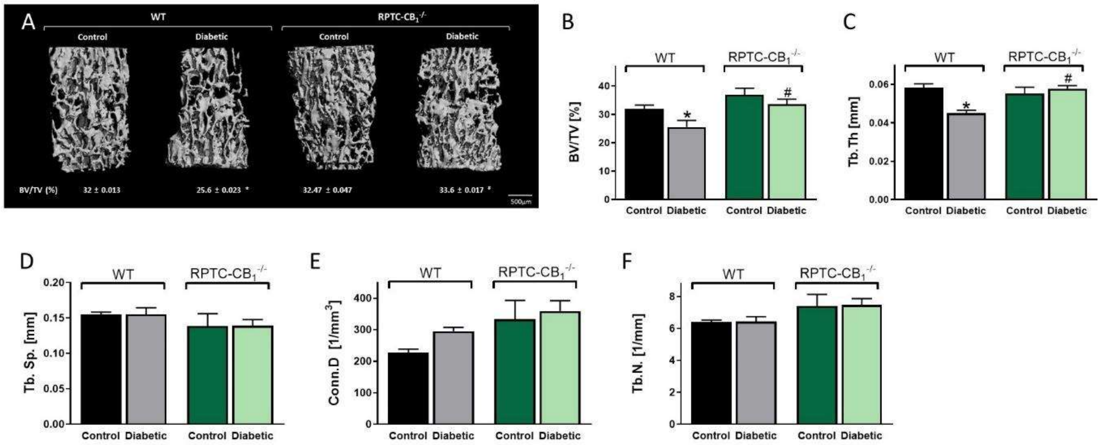

Supplementary Figure 3. Effect of CB1R nullification in RPTCs on vertebral parameters under hyperglycemic conditions. 3D images of the vertebrae of mice with median BV/TV values (**A**), Analysis of the bone volume density of L3 verterbrae (BV/TV) values (B), L3 trabecular thickness (Tb.Th; **C**), L3 trabecular spacing (Tb.Sp; **D**), L3 connectivity density (Conn.D; **E**), and L3 trabecular number (Tb.N; **F**). Data represent the mean ± SEM from 8-13 animals per group. **P*<0.05 vs. non-diabetic WT control mice, #*P*<0.05 vs. diabetic WT mice.

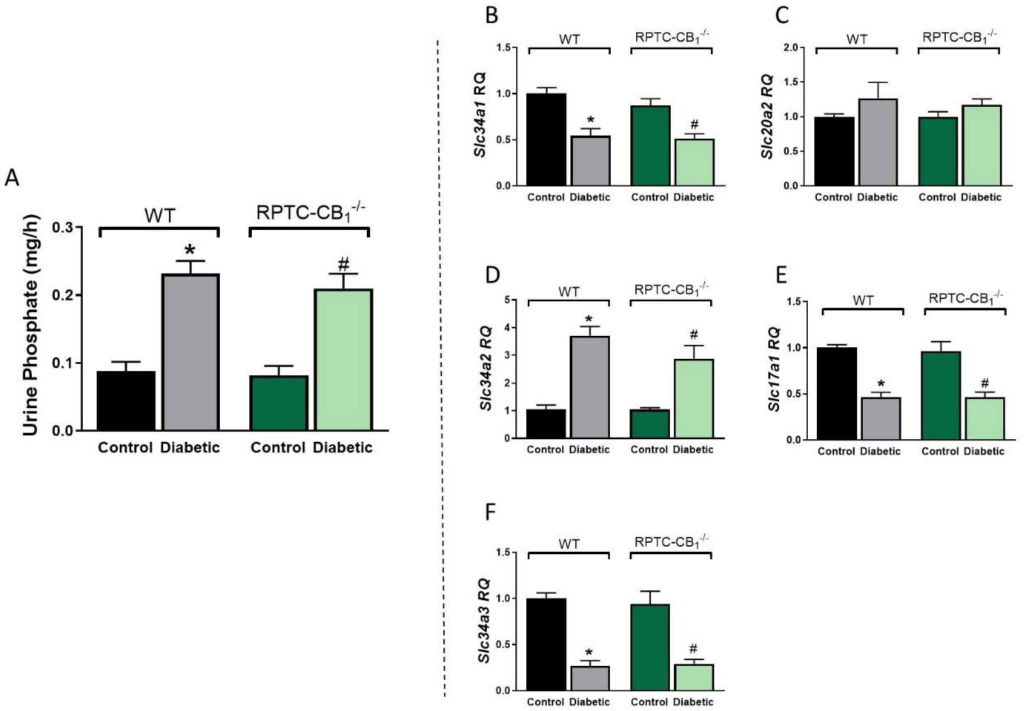

Supplementary Figure 4. Effect of CB$_1$R nullification in RPTCs on phosphate renal reabsorption. Urine Phosphate Levels (**A**). Analysis of gene expression levels of various renal phosphate transporters (**B-F**). Data represent the mean ± SEM from 8-13 animals per group. **P*<0.05 vs. non-diabetic WT control mice, #*P*<0.05 vs. nondiabetic RPTC-CB$_1$R KO mice.

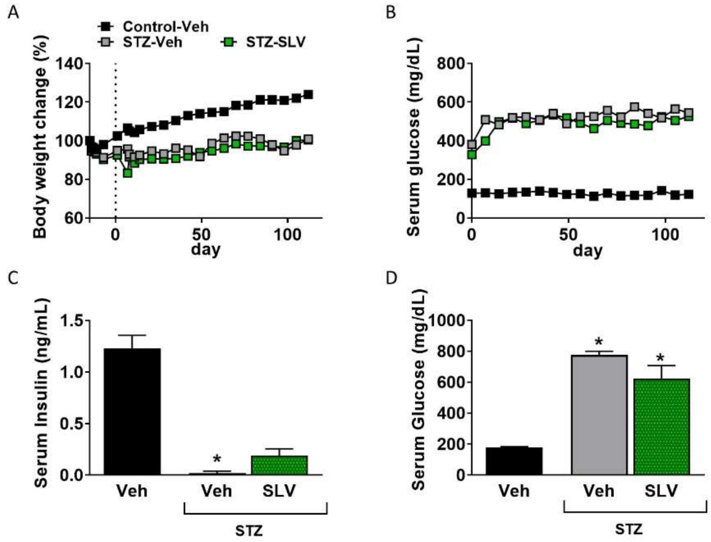

Supplementary Figure 5. The effect of CB1R blockade on body weight and glucose homeostasis in T1D. Body weight and serum glucose changes in control animals treated orally with Veh in comparison to diabetic mice treated orally with SLV-319 (3 mg/kg), or Veh for 16 weeks (**A, B**). Serum insulin (**C**) and glucose levels (**D**) after 16 weeks of treatment. Data represent the mean ± SEM from 8-10 mice per group. **P<0.05* relative to the corresponding control group treated with Veh.

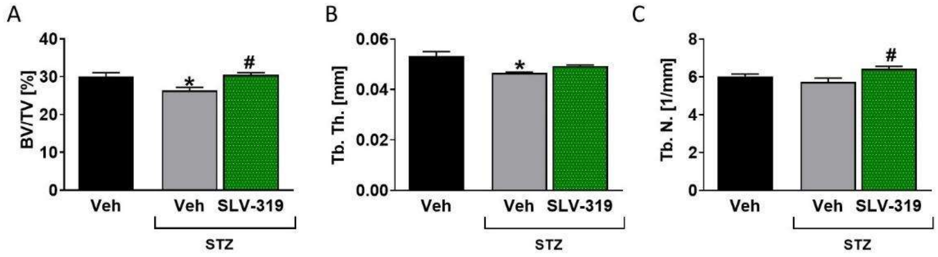

Supplementary Figure 6. Chronic CB1R blockade prevents diabetes-induced vertebral bone loss. Analysis of the bone volume density of L3 verterbrae (BV/TV) values (**A**). L3 trabecular thickness (Tb.Th; **B**), and L3 trabecular number (Tb.N; **C**). Data represent the mean ± SEM from 8-10 mice per group. **P*<0.05 relative to the corresponding control group treated with Veh, #*P*<0.05 relative to the corresponding STZ group treated with Veh.
